# Innovative Carbon Ball Frameworks: Elevating Energy Storage Performance and Enhancing CO_2_ Capture Efficiency

**DOI:** 10.3390/polym16040516

**Published:** 2024-02-14

**Authors:** Thirukumaran Periyasamy, Shakila Parveen Asrafali, Seong-Cheol Kim, Jaewoong Lee

**Affiliations:** 1 Department of Fiber System Engineering, Yeungnam University, Gyeongsan 38541, Republic of Korea; 2 School of Chemical Engineering, Yeungnam University, Gyeongsan 38541, Republic of Korea

**Keywords:** porous carbon, heteroatom doping, CO_2_ adsorption, supercapacitor application

## Abstract

A novel porous carbon, derived from polybenzoxazine and subjected to hydrogen peroxide treatment, has been meticulously crafted to serve dual functions as a supercapacitor and a CO_2_ capture material. While supercapacitors offer a promising avenue for electrochemical energy storage, their widespread application is hampered by relatively low energy density. Addressing this limitation, our innovative approach introduces a three-dimensional holey carbon ball framework boasting a hierarchical porous structure, thereby elevating its performance as a metal-free supercapacitor electrode. The key to its superior performance lies in the intricate design, featuring a substantial ion-accessible surface area, well-established electron and ion transport pathways, and a remarkable packing density. This unique configuration endows the holey carbon ball framework electrode with an impressive capacitance of 274 F g^−1^. Notably, the electrode exhibits outstanding rate capability and remarkable longevity, maintaining a capacitance retention of 82% even after undergoing 5000 cycles in an aqueous electrolyte. Beyond its prowess as a supercapacitor, the hydrogen peroxide-treated porous carbon component reveals an additional facet, showcasing an exceptional CO_2_ adsorption capacity. At temperatures of 0 and 25 °C, the carbon material displays a CO_2_ adsorption capacity of 4.4 and 4.2 mmol/g, respectively, corresponding to equilibrium pressures of 1 bar. This dual functionality renders the porous carbon material a versatile and efficient candidate for addressing the energy storage and environmental challenges of our time.

## 1. Introduction

Electrochemical capacitors (ECs), commonly referred to as supercapacitors, have emerged as a compelling and innovative technology in the realm of energy storage and portable power applications. Although ECs offer superior power density and cycle life compared to traditional batteries, a significant challenge persists in their lower energy density, trailing behind batteries by at least one order of magnitude [1,2,3]. The linchpin to enhancing an EC’s overall performance lies in the intricate realm of electrode materials, necessitating the development of materials that can boost energy density without compromising power density or cycle life [4,5,6,7]. The pursuit of high-performance EC electrodes revolves around achieving several crucial attributes, including high electrical conductivity, an abundance of ion-accessible surface area, rapid ionic transport, and robust electrochemical stability. Carbon-based porous materials have emerged as the frontrunners in this quest, presenting a promising class of EC electrode materials [8,9,10]. Currently, porous activated carbon electrodes dominate the landscape of ECs, showcasing a gravimetric capacitance ranging from 80 to 120 F g^−1^ and a stack energy density of 4–5 W h kg^−1^. Despite these notable figures, they still fall significantly short of the energy density exhibited by lead–acid batteries, which range from 25 to 35 W h kg^−1^ [11,12].

In recent developments, graphene has captured the attention of researchers as a potential game-changer in the realm of EC electrode materials. Its appeal stems from its extraordinary intrinsic electrical conductivity, mechanical flexibility, vast theoretical surface area (2630 m^2^ g^−1^), and a theoretical gravimetric capacitance of approximately 550 F g^−1^. However, a significant drawback impeding graphene’s widespread adoption is its tendency to restack into graphite-like formations, resulting in unsatisfactory gravimetric capacitances [7,9].

To address these challenges, innovative approaches have been explored. Traditional activation methods and laser-scribed graphene with an open porous structure have been employed to enhance the accessible surface area and gravimetric capacitance. However, the focus is shifting towards considering volumetric performance, recognizing the trade-off relationship between gravimetric and volumetric capacitances in most electrode designs. Highly porous electrodes offer large specific surface areas for high gravimetric capacitance but often result in lower volumetric capacitance due to their lower packing density [6,11]. The intricate relationship between energy density and other crucial performance metrics necessitates a holistic approach to material development. Researchers are actively exploring avenues to overcome the limitations of current materials and unlock the full potential of ECs [7,10]. One promising strategy involves the design and synthesis of advanced carbon-based materials that go beyond traditional activated carbon. These novel materials aim to address the current energy density bottleneck while preserving the advantageous properties of high electrical conductivity, large surface area, and electrochemical stability [13].

In the midst of the global drive towards sustainable energy solutions, prompted by both the intensifying energy crisis and growing environmental apprehensions, advanced materials capable of bolstering energy storage efficiency and curbing carbon emissions are imperative. Porous carbon, with its expansive specific surface area, minimal resistivity, and eco-friendly attributes, has not only gained prominence in diverse fields like wastewater purification and catalyst carriers but has also emerged as a pivotal player in electrochemical energy storage. The significance of multi-stage pore structures within carbon materials becomes glaringly evident in energy storage applications, where such structures contribute to heightened charge/discharge rates, increased specific capacitance, and enhanced cycle performance. Moreover, the potential of hierarchical nano-pore porous carbon materials in CO_2_ capture is noteworthy, as their structure optimizes physical adsorption pathways and mass transfer kinetics [14,15,16]. One viable solution to enhance the specific properties of porous carbon materials lies in heteroatom doping, with nitrogen doping standing out as particularly promising. Nitrogen doping introduces a range of advantageous modifications to porous carbon, including a significant reduction in resistivity, improved carbon wettability, and heightened basicity. These alterations result in materials that exhibit improved or entirely new properties, making them highly desirable for a variety of applications, spanning from CO_2_ adsorption to supercapacitors and electrocatalytic reactions [13,15].

In this context, the work of Sevilla and colleagues stands out, as their studies have demonstrated the potential of nitrogen-doped polypyrrole-based porous carbons for high-capacity CO_2_ capture. By strategically incorporating nitrogen into the carbon matrix, these researchers have succeeded in overcoming some of the traditional limitations associated with porous carbon materials. The resulting nitrogen-doped porous carbons exhibit enhanced performance in terms of CO_2_ adsorption capacity, offering a promising avenue for mitigating greenhouse gas emissions [17,18,19]. Expanding on the impact of nitrogen doping, it is crucial to highlight its role in mitigating resistivity within porous carbon materials. The reduction in resistivity is a critical factor in improving the overall efficiency of energy storage systems. High resistivity can impede the flow of electrons within the material, leading to energy losses and decreased performance. Nitrogen doping addresses this issue by facilitating better electron transport, thereby enhancing the conductivity of porous carbon materials. This improvement is instrumental in achieving faster charge/discharge rates, a key parameter in the efficacy of energy storage devices [20].

Moreover, the influence of nitrogen doping on carbon wettability is a noteworthy aspect of its impact on porous carbon materials. The introduction of nitrogen alters the surface chemistry of the carbon material, leading to changes in its interaction with liquid electrolytes. This modification can result in improved wetting properties, facilitating better electrolyte penetration into the porous structure. Enhanced wettability contributes to more efficient electrochemical reactions, further improving the performance and longevity of energy storage devices [21,22,23]. Another significant advantage of nitrogen doping in porous carbon materials is its effect on basicity. Nitrogen-doped carbons often exhibit increased basicity, which can be advantageous in various applications. In the realm of CO_2_ capture, for example, basic sites on the material’s surface can facilitate the chemisorption of carbon dioxide, enhancing the overall capture efficiency. This property is of paramount importance as the world seeks effective strategies to combat climate change and reduce greenhouse gas emissions [15,17,24].

In this context, the work described here evidences the synthesis of porous carbon along with heteroatom doping containing nitrogen and oxygen. This doping could be performed easily from a polybenzoxazine source. Furthermore, the versatility of nitrogen-doped porous carbon extends beyond CO_2_ capture to include supercapacitors and electrocatalytic reactions. The improved conductivity and wettability, coupled with enhanced basicity, make these materials highly suitable for energy storage and conversion applications. Supercapacitors, known for their rapid charge/discharge capabilities, can benefit significantly from the attributes conferred by nitrogen doping. The increased capacitance and conductivity contribute to elevated energy storage and release rates, aligning with the growing demand for efficient energy storage solutions in various industries.

## 2. Materials and Methods

### 2.1. Materials

4-aminophenol and paraformaldehyde were purchased from Sigma-Aldrich (St. Louis, MO, USA). Hydrogen peroxide (H_2_O_2_), sodium hydroxide (NaOH), dimethyl sulfoxide (DMSO), ethanol, polyvinylidene fluoride (PVDF), and N, N-dimethylformamide (DMF) were purchased from Duksan Chemicals Co., Ltd. (Gyeonggi-do, Republic of Korea). All chemicals were used without further purification.

### 2.2. Methods

#### Synthesis of Polybenzoxazine Carbon Balls (CB) and Hydrogen Peroxide-Treated Carbon Balls (H-CB)

The synthesis of benzoxazine monomer commenced with the preparation of a 100 mL aqueous–ethanol solution, achieved by combining ethanol and distilled water in a 1:1 ratio. To this mixture, 7.33 g of 4-aminophenol was meticulously introduced under continuous stirring at 30 °C until complete dissolution was achieved. After a duration of 20 m, 12.5 mL of formaldehyde was judiciously added, initiating polymerization that continued for 1 h under constant stirring at 30 °C. Subsequently, the reaction temperature experienced an elevation to 75 °C, and the system was stirred at this temperature for an additional 4 h. The resulting 4-aminophenol-formaldehyde resin polymer balls were separated through centrifugation and subsequently dried at 100 °C overnight. For the generation of nitrogen-doped carbon balls, these resin polymer balls underwent carbonization under a nitrogen atmosphere in a tube furnace. The process involved a heating rate of 1 °C/min, reaching 350 °C, and a dwell time of 2 h, followed by a resumption of the heating rate to 800 °C with a subsequent dwell time of 4 h. The resultant carbon materials were denoted as CB.

**Scheme 1:**
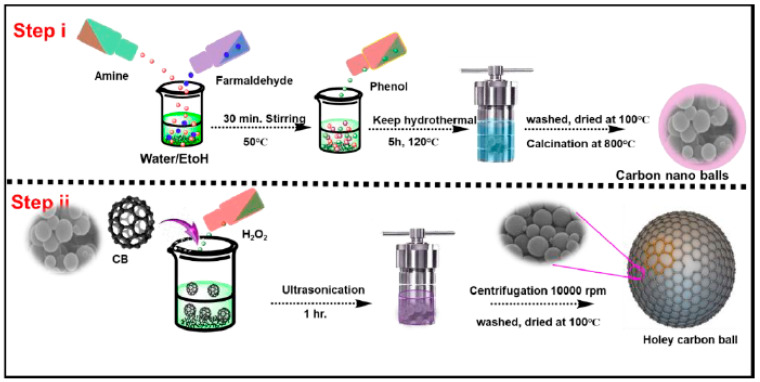
Schematic illustration showing the synthesis procedure of CB and H-CB.

The preparation of H-CB was initiated by introducing a 5 mL diluted H_2_O_2_ aqueous solution (3% H_2_O_2_) into 25 mL of 5 mg CB aqueous dispersion within a 100 mL Teflon-lined autoclave. This mixture was sealed and subjected to heating at 180 °C for 6 h, followed by natural cooling to room temperature. The as-prepared H-CB was then extracted, undergoing thorough washing with pure water to eliminate any residual impurities in preparation for subsequent experiments. This meticulous process ensures the controlled synthesis of benzoxazine monomer and its derivatives with tailored properties for diverse applications (Scheme 1).

## 3. Results

### 3.1. Structural Analysis of Carbon Balls (CB) and Hydrogen Peroxide-Treated Carbon Balls (H-CB)

**Figure 1:**
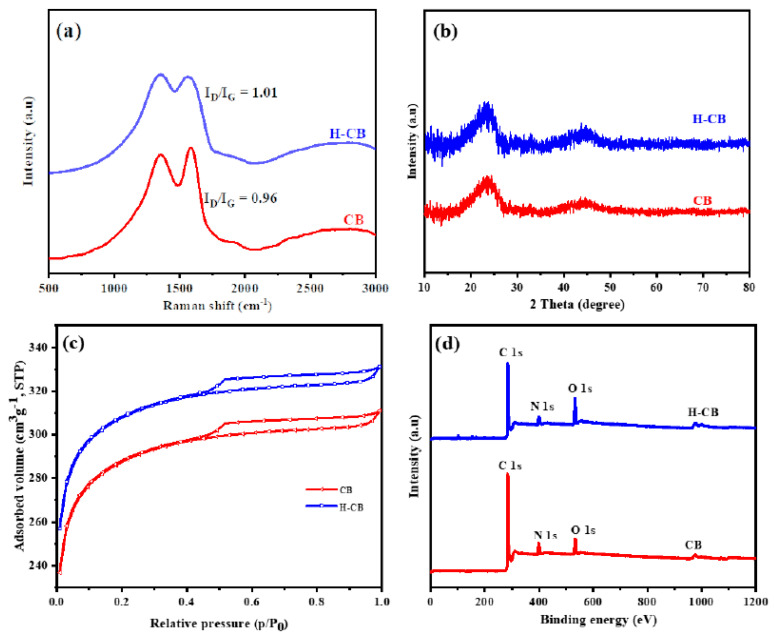
(**a**) Raman spectra, (**b**) XRD, (**c**) N_2_ adsorption isotherms, and (**d**) XPS spectra of CB and H-CB.

The structural features and graphitic attributes of carbon balls (CB) and carbon balls treated with hydrogen peroxide (H-CB) were examined through Raman and XRD analyses. Figure 1a depicts the Raman spectra of CB and H-CB carbons. Both samples exhibit prominent peaks at 1354 and 1581 cm^−1^, corresponding to the ‘D’ and ‘G’ bands, respectively. Notably, H-CB displays an intensified ‘D’ band, indicating a higher degree of disorder in the carbon structure due to the presence of an increased number of heteroatoms. The I_D_/I_G_ ratio, representing the degree of graphitization, was found to be 0.96 for CB and 1.01 for H-CB, indicating increased disorder upon hydrogen peroxide treatment [25,26].

Figure 1b showcases the XRD patterns of CB and H-CB carbons. Two broad peaks at 2θ = 24.2 and 44.6°, corresponding to the diffraction of (002) and (100) planes, respectively, typify graphitic carbon materials. Interestingly, H-CB exhibits a gradual reduction in peak intensity at 2θ = 24.2° (100), indicating an increased degree of graphitization. Utilizing Bragg’s equation, the calculated d-spacing value for graphitic carbon was determined to be 0.37 nm. This value surpasses that of conventional graphite, suggesting that these materials may contribute to enhanced supercapacitor capacity when employed as electrodes [27]. The results prove that there is a positive impact of hydrogen peroxide treatment on the structural order and graphitic properties of carbon balls. The increased disorder observed in H-CB due to H_2_O_2_ treatment holds promise for applications in supercapacitors, where enhanced structural characteristics contribute to improved performance as electrodes.

The nitrogen adsorption–desorption isotherms depicted in Figure 1c illustrate the distinctive characteristics of activated carbons CB and H-CB. Both exhibit type IV isotherms with analogous hysteresis loops in the meso-pore range [28]. Micro-pore adsorption, indicated by a sharp rise in N_2_ isotherms at a relative pressure below 0.1, intensifies with elevated activation temperature. Notably, H-CB displays a broader micro-pore size distribution compared to CB, attributed to the higher activation temperature employed. The progressive augmentation of adsorption isotherms, observed with increasing relative pressure up to P/P_0_ = 0.9, signifies the gradual adsorption of N_2_ molecules on the meso-pores. Beyond a relative pressure of 0.9 (P/P_0_), a pronounced upswing in the isotherms suggests the presence of macro-pores. The BET isotherms yield specific surface areas of 784.3 and 1242.7 m^2^ g^−1^ for CB and H-CB, respectively [1]. Interestingly, the hydrogen peroxide treatment results in a slightly enlarged average meso-pore size, indicating that severe activation conditions, coupled with H_2_O_2_ etching, contribute to meso-pore expansion. The nitrogen adsorption–desorption isotherms provide valuable insights into the pore characteristics of CB and H-CB, highlighting the impact of hydrogen peroxide treatment on micro- and meso-pore structures.

**Figure 2:**
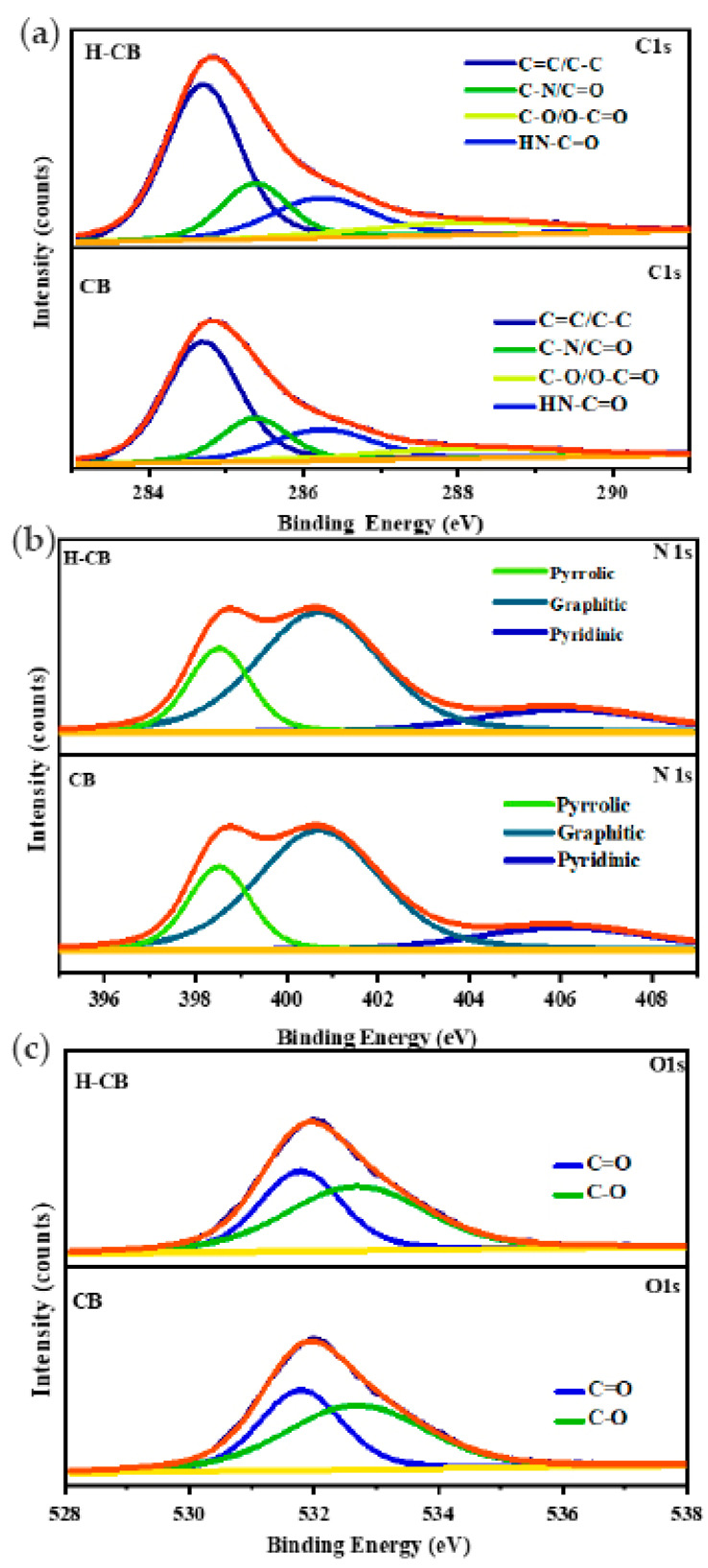
XPS spectra of CB and H-CB showing the deconvoluted spectrum for C 1s, N 1s, and O 1s.

Figure 1d depicts the X-ray photoelectron spectroscopy (XPS) survey scans of carbon ball (CB) and hydrogen peroxide-treated carbon ball (H-CB) materials. The obtained spectra exhibit distinct photoelectron peaks for carbon (C), nitrogen (N), and oxygen (O) at approximately 285.8, 399.4, and 532.1 eV, respectively. Subsequent analyses focused on the C 1s, N 1s, and O 1s signals, revealing intricate details as illustrated in Figure 2a–c. The deconvolution of the C 1s spectrum (Figure 2a) unveiled four distinctive peaks: the first at 284.7 eV corresponding to hydrocarbon chains (C=C/C–C), the second at 285.4 eV associated with carbon atoms in the C-N bond, the third at 286.3 eV attributed to carbon atoms bonded with O and N (HN–C=O) groups, and the fourth at 287.5 eV indicative of O–C=O/C=N/C–OH groups.

Moving to Figure 2b, an exploration of nitrogen species within activated porous carbons is presented. Three discernible nitrogen species are identified at 399.1 eV (pyrrolic nitrogen), 400.3 eV (quaternary nitrogen), and 405.4 eV (pyridinic nitrogen). Notably, the activated porous carbons exhibit a higher concentration of pyridinic and pyrrolic nitrogen species, potentially stemming from the polybenzoxazine source. These nitrogen species exhibit electrochemical activity in acidic aqueous solutions, enhancing capacitance. Conversely, quaternary nitrogen species within the carbon matrix promote electron transfer, thereby improving conductivity. The superior conductivity of the H-CB sample, attributed to its higher degree of graphitization and thermally stable quaternary nitrogen groups, highlights its potential for enhanced electrical performance [29,30].

The O 1s spectrum, presented in Figure 2c, is deconvoluted into two distinct peaks at 532.1 and 533.2 eV, revealing the presence of quinone (Ph=O), phenolic and hydroxyl or ether (C–O–C), chemisorbed oxygen, or water functional groups. Among these oxygen functional groups, quinone groups in the carbon matrix are identified as electrochemically inactive in reversible redox reactions in an alkaline medium. Conversely, phenolic hydroxyls (via reduction) and hydroxyls or ethers (via deprotonation) exhibit quasi-reversible pseudocapacitances. Consequently, the meticulously prepared H-CB material, enriched with phenolic hydroxyls or ether oxygens and carbonyl oxygens, demonstrates the ability to generate high pseudo-capacitance in an acidic alkaline electrolyte. This electrochemical behavior holds promise for applications demanding efficient and reversible charge storage, underscoring the significance of tailored material synthesis for advanced energy storage systems.

### 3.2. Morphology Analysis of Carbon Balls (CB) and Hydrogen Peroxide-Treated Carbon Balls (H-CB)

**Figure 3:**
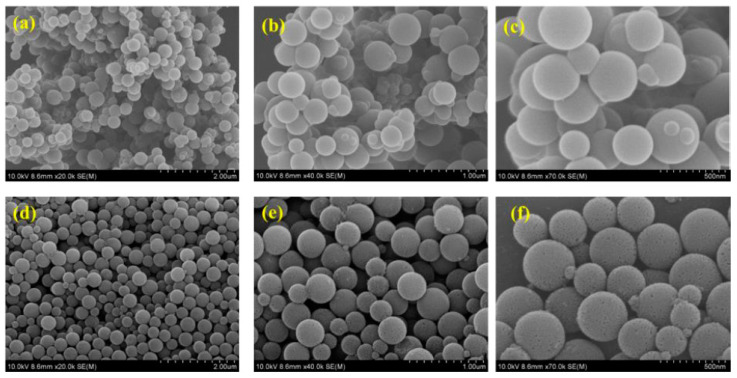
SEM images of CB (**a**–**c**) and H-CB (**d**–**f**).

The surface morphology of both pristine carbon balls (CBs) and hydrogen peroxide-treated carbon balls (H-CBs) was scrutinized through field emission scanning electron microscopic (FESEM) analysis. Figure 3a–c depict the FESEM images of pristine CBs at varying magnifications. These images reveal that the particles exhibit a ball-like and monodispersed nature, showcasing a smooth morphology. A closer inspection at high magnification unveils a predominantly non-porous structure composed of numerous nanoparticles, creating a micro-porous surface. The average diameter of the pristine CBs measures approximately 100–300 nm.

Upon subjecting the CBs to hydrogen peroxide treatment, the surface of the resulting H-CB (Figure 3d–f) becomes noticeably rougher, indicating the development of a porous framework through the action of H_2_O_2_ and the activation process. Remarkably, the carbon maintains its porous and spherical structure, but the simultaneous processes of exfoliation and excavation leave the interior hollow. The favorable hierarchical porous and hollow structures of H-CB contribute to enhanced CO_2_ adsorption and superior supercapacitance performance. These structures facilitate the adsorption and diffusivity of CO_2_, ensuring faster CO_2_ adsorption. Additionally, the hollow structure reduces transport resistance, enhancing diffusion properties. In the context of supercapacitance, the porous channels enable faster mass diffusion and transport, creating a shorter diffusion pathway for high supercapacitance performance. The hollow interior also provides more accessible reactive sites, facilitating ion diffusion.

**Figure 4:**
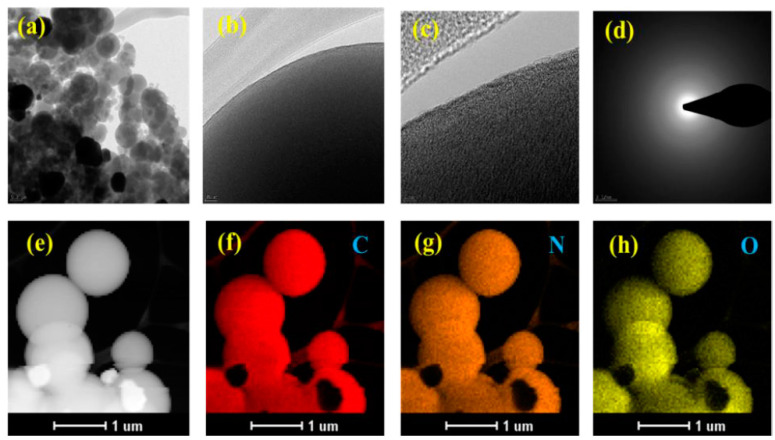
TEM image with corresponding SAED pattern and elemental mapping of H-CB.

The importance of the hierarchical pore structure in both CO_2_ adsorption and supercapacitance is further emphasized in the transmission electron microscopy (TEM) images in Figure 4a–c. The surface of H-CB appears rougher, with evidence of exfoliation into thin sheets. These thin sheets exhibit microporous features, as illustrated in the higher magnification TEM images. Additionally, an increased quantity of exfoliated sheets and a hollow interior are observed in H-CB. Figure 4c shows uniform meso-pores, likely formed due to the pore-widening effect induced by H_2_O_2_ treatment.

The selected area electron diffraction (SAED) pattern in Figure 4d distinctly demonstrates the amorphous nature of H-CB. Furthermore, energy-dispersive X-ray spectroscopy (EDS) maps confirm the uniform distribution of all elements, including C, N, and O. The heteroatoms N and O are uniformly embedded within the carbon framework (Figure 4e–h). The amalgamation of these initial characterizations provides robust evidence that H-CB has been successfully synthesized with the desired morphology, positioning it as an ideal electrode material for electrochemical applications and CO_2_ adsorption. The surface modifications induced by hydrogen peroxide treatment result in hierarchical porous and hollow structures in carbon balls, enhancing their suitability for both CO_2_ adsorption and supercapacitor applications. The detailed microscopic analyses presented in FESEM and TEM images, along with SAED and elemental mapping data, collectively affirm the successful synthesis of H-CB with the desired morphology, marking a significant advancement in materials tailored for electrochemical and environmental applications.

### 3.3. Electrochemical Performance of CB and H-CB

**Figure 5:**
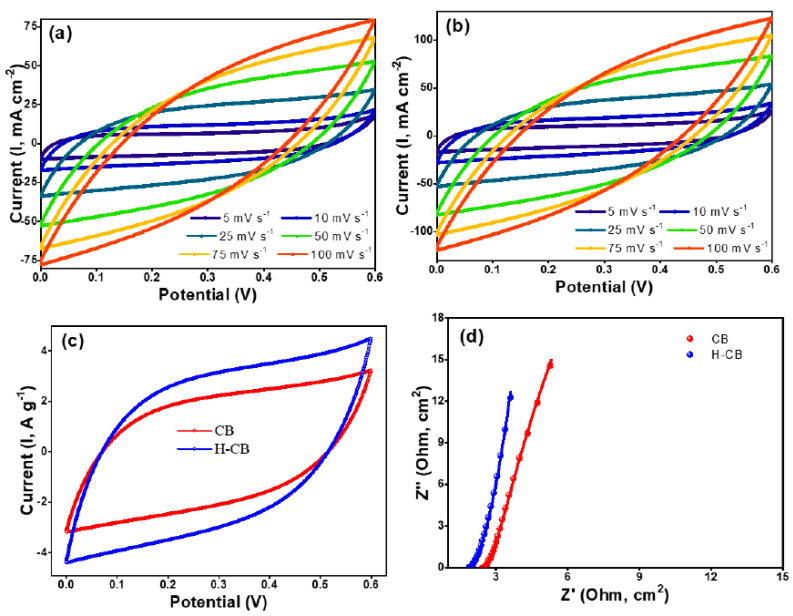
Electrochemical studies showing the CV (**a**–**c**) and Nyquist plots of CB and H-CB (**d**).

The investigation of electrochemical performance of the materials is crucial for optimizing the efficiency of energy storage devices. This study delves into the enhanced electrochemical characteristics of H-CB electrodes compared to CB electrodes. Comprehensive analyses, including cyclic voltammetry, electrochemical impedance spectroscopy, galvanostatic charge/discharge studies, and cycling tests were conducted to understand the distinct features contributing to the improved performance of H-CB. Cyclic voltammetry (CV) analysis was carried out at various scan rates to evaluate the electrochemical behavior of CB and H-CB electrodes. At a high scan rate of 100 mV s^−1^, H-CB exhibited significantly enhanced performance compared to CB, as depicted in Figure 5a,b. The quasi-rectangular CV curves at different scan rates, particularly the nearly ideal shape at 25 mV s^−1^, indicate the superior electrical-double-layer capacitive behavior of H-CB (Figure 5c). The observed weak pair of broad peaks in the CV curve suggest a minor contribution from the redox reaction of residual oxygen-containing groups on the carbon of H-CB [31,32,33].

To further probe the ion transport properties within H-CB, electrochemical impedance spectroscopy (EIS) was employed. The Nyquist plots (Figure 5d) revealed a nearly ideal capacitive behavior in the low-frequency regime, with a transition to a 45° Warburg region and a semicircle in the high-frequency regime. The H-CB electrodes demonstrated a shorter 45° region and a smaller diameter semicircle, indicating lower charge transfer resistance and more efficient electrolyte diffusion compared to CB. Extrapolation of the plots yielded equivalent series resistance (ESR) values of 1.5 Ω for H-CB and 2.4 Ω for CB.

**Figure 6:**
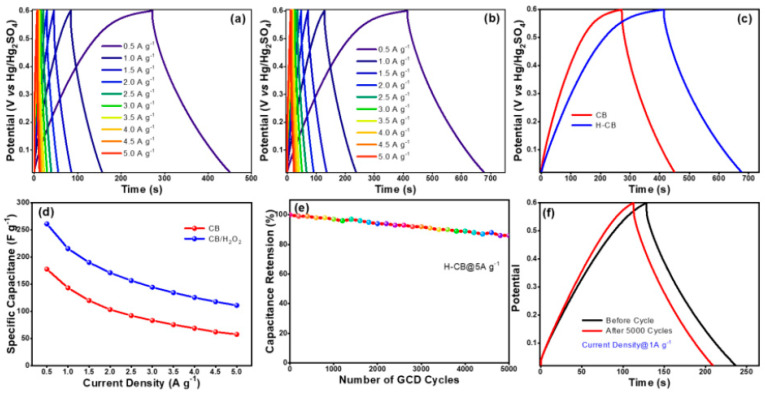
GCD of CB and H-CB (**a**–**c**), specific capacitance calculated from GCD (**d**), cycling stability of H-CB at 5A g^−1^ (**e**) and GCD for 1st and 5000th of H-CB (**f**).

The galvanostatic charge/discharge studies conducted up to a high current density of 5 A g^−1^ (Figure 6a,b) illustrated the superior electrochemical performance of H-CB over CB. The nearly triangular charge/discharge curves for H-CB indicated enhanced energy storage capabilities (Figure 6c). Furthermore, the voltage drop analysis revealed a smaller IR drop (0.15 V) for H-CB compared to CB (0.27 V), resulting in ESR values of 1.5 Ω and 2.4 Ω, respectively [34,35,36,37]. The specific capacitance values derived from the charge/discharge curves demonstrated the remarkable performance of H-CB electrodes. At a current density of 0.5 A g^−1^, H-CB exhibited an ultrahigh specific capacitance of 274 F g^−1^, surpassing CB electrodes, which showed a capacitance of about 186 F g^−1^ at the same current density (Figure 6d). Even at a higher current density of 5 A g^−1^, H-CB retained approximately 83% of its capacitance, showcasing its excellent rate capability (Figure 6e).

The cycling test results exhibited 85% capacitance retention over 5000 cycles at a high current density of 1 A g^−1^ (Figure 6f), underscoring the exceptional electrochemical stability of H-CB electrodes. This longevity in performance positions H-CB as a promising material for energy storage applications, offering not only higher specific capacitance but also improved rate capability and capacitance retention at high charging/discharging rates. The comprehensive electrochemical analyses presented in this study highlight the superior performance of H-CB electrodes compared to CB electrodes. The unique hierarchical porous network of H-CB contributes to its enhanced ion-accessible surface area and efficient ion transport, leading to higher specific capacitance, improved rate capability, and outstanding capacitance retention. These findings underscore the potential of H-CB as a promising material for advanced energy storage devices [38,39,40,41].

### 3.4. CO_2_ Adsorption Studies

**Figure 7:**
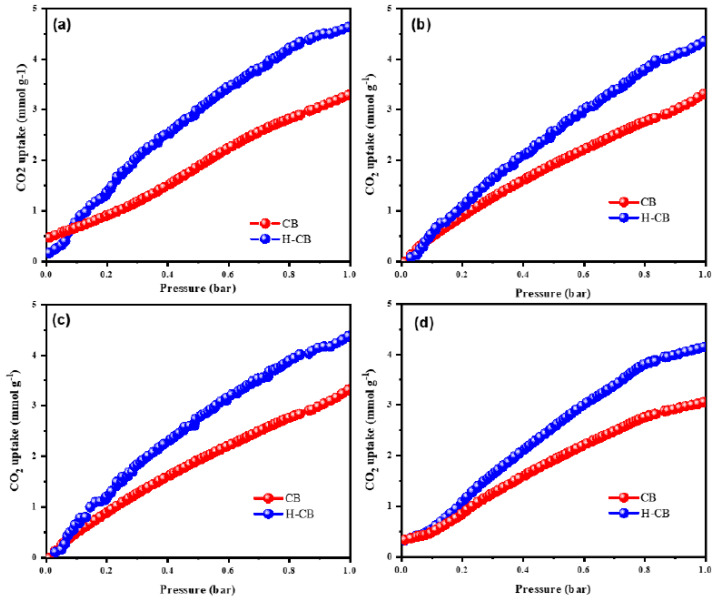
CO_2_ adsorption (**a**,**b**) and desorption (**c**,**d**) isotherms for the CB and H-CB measured at 0 and 25 °C.

The adsorption capacity of the synthesized porous materials for CO_2_ is intricately linked to two key factors: the nitrogen content and the pore structure’s availability. The nitrogen atom plays a crucial role in interacting with and retaining CO_2_, while the pores, spanning micro-, meso-, and macropores, facilitate the diffusion and transmission of the gas. The CO_2_ adsorption occurs by both physisorption and chemisorption. In case of physisorption, the presence of active surface area of the material with micro-, meso-, and macro-porous structure makes the CO_2_ molecules get adsorbed on the surface of the porous carbon material through van der Waals attraction, whereas chemisorption takes place through chemical bonding between the CO_2_ molecules and hetero-doped part of the porous carbon materials. Figure 7a–d represent the CO_2_ adsorption and desorption isotherms for CB and H-CB, respectively, at 0 and 25 °C. The data presented in Figure 7a,b illustrate that CB and H-CB exhibit CO_2_ adsorption capacities of 3.2 and 4.4 mmol g^−1^ at 273 K and 3.0 and 4.2 mmol g^−1^ at 298 K, respectively. Similar results were obtained with Li et al., where they found that the activated expanded vermiculite-derived silica along with PEI (AEVP50 adsorbent) exhibited a high CO_2_ uptake of 2.21 mmol g^−1^ at 75 °C. Shi et al. synthesized activated carbon materials from poly(E-lyme) (from biobased benzoxazines synthesized from amino acid methyl ester and disphenolic acid methyl ester/eugenol) showing a remarkably high CO_2_ adsorption capacity of 5.82 mmol g^−1^ and exhibiting high surface area of up to 1194 m^2^ g^−1^. Interestingly, despite similar nitrogen content in CB and H-CB, the latter demonstrates a significantly higher CO_2_ adsorption capacity. This enhancement can be attributed to the hierarchical porous structure inherent in H-CB, encompassing micro-, meso-, and macro-pores [42]. The hierarchical nature of the pores in H-CB expedites the diffusion of CO_2_ from the material’s surface into active sites through a combination of chemical bonding and physical adsorption, thereby augmenting the overall CO_2_ adsorption capacity. In contrast, CB, possessing solely micro-pores, impedes the efficient diffusion of CO_2_ from the surface to the interstices, resulting in a reduced adsorption capacity. It is noteworthy that in CB, the CO_2_ adsorption capacity is solely reliant on nitrogen content, whereas in H-CB, both the nitrogen content and the hierarchical porous structure contribute synergistically to the heightened CO_2_ adsorption [43,44,45].

The intricate CO_2_ adsorption process involves three distinct phenomena. Firstly, the micro-pores on the carbon surface play a pivotal role in trapping CO_2_ gas. Secondly, the nitrogen content, comprising pyrrolic-N and pyridinic-N, acts as an anchor for adsorbed CO_2_. Lastly, the meso-pores serve as conduits, facilitating the diffusion of CO_2_ from the surface to the active sites. At a higher temperature of 298 K, the CO_2_ adsorption capacity for both materials experiences a decrease. This phenomenon can be attributed to the exothermic nature of CO_2_ adsorption, favoring lower temperature conditions. A similar trend is observed in CO_2_ desorption at 273 and 298 K, as depicted in Figure 7c,d. The interplay between temperature and the adsorption–desorption processes underscores the intricate thermodynamic aspects governing CO_2_ interactions with porous materials. This study underscores the critical role of nitrogen content and pore structure in dictating the CO_2_ adsorption capacity of porous materials. The hierarchical porous structure in H-CB, in conjunction with nitrogen content, emerges as a decisive factor in enhancing CO_2_ adsorption. The multifaceted adsorption process involving micro-pores, nitrogen content, and meso-pores contributes to the nuanced behavior observed at varying temperatures, shedding light on the complex dynamics of CO_2_ adsorption on porous carbon materials [46,47].

## 4. Conclusions

Our innovatively crafted porous carbon material, derived from polybenzoxazine and treated with hydrogen peroxide, represents a revolutionary advancement in the realms of electrochemical energy storage and environmental preservation. The distinctive three-dimensional porous structure of the carbon material resembles a network of interconnected carbon balls. This breakthrough is pivotal in reshaping the landscape of energy storage technologies. Furthermore, the material’s versatility extends beyond energy storage to environmental conservation. Notably, the porous carbon material exhibits an extraordinary ability to capture carbon dioxide (CO_2_), accentuating its multifaceted utility in addressing pressing environmental challenges. The impressive capacitance of 274 F g^−1^ underscores its efficacy as a high-performance supercapacitor material. In addition to its electrochemical prowess, the material demonstrates exceptional CO_2_ adsorption capacities at temperatures of 0 and 25 °C, registering 4.4 and 4.2 mmol/g, respectively, at equilibrium pressures of 1 bar. This dual functionality positions the porous carbon material as a versatile and efficient solution to the contemporary challenges of energy storage and environmental sustainability. By seamlessly integrating cutting-edge technology with environmental consciousness, our porous carbon material emerges as a beacon of hope in the quest for sustainable and impactful advancements.

## Data Availability

The data presented in this study are available in the article.
